# Barcoding and species delimitation of Iranian freshwater crabs of the Potamidae family (Decapoda: Brachyura)

**DOI:** 10.1038/s41598-022-12335-w

**Published:** 2022-05-18

**Authors:** Kamran Rezaei Tavabe, Mina Tavana, Ali Reza Mirvaghefi, Arash Jouladeh-Roudbar, Paniz Rahimi, Ignacio Doadrio, Hamid Reza Ghanavi

**Affiliations:** 1 Fisheries Department, University of Tehran, Karaj, Iran; 2 Museo Nacional de Ciencias Naturales, MNCN-CSIC, Madrid, Spain; 3 Lund University, Ecology Building, Sölvegatan 37, 22362 Lund, Skåne Sweden

**Keywords:** Ecology, Zoology, Ecology, Environmental sciences, Limnology

## Abstract

Freshwater ecosystems are under multiple threats in modern times such as water extraction for human consumption, industries and agricultural activities, water contamination and habitat destruction for example. At the same time the biodiversity of these ecosystems are often poorly studied, especially in arid countries such as Iran. In this work, we study one of the ecologically important members of Iranian freshwater fauna, freshwater crab species of the genus *Potamon*. Here, we barcoded the different populations occurring in the country and delimited the species to allow for a better understanding of their distribution and taxonomy. In this study, we evaluated the taxonomical statues of *Potamon* species in Iran using genetic data. In addition, we created the first barcoding reference for Iranian freshwater crabs, which is an important resource for future environmental and conservation studies.

## Introduction

Freshwater crabs (Crustacea: Decapoda: Brachyura Linnaeus, 1758) are different groups of Eubrachyurans, comprising approximately 20% of the biodiversity of Brachyuran. They are classified into three superfamilies: Potamoidea (Ortmann, 1896), Gecarcinucoidea Rathbun, 1904 and Trichodactyloidea (H. Milne Edwards, 1853)^1^. A new superfamily Pseudothelphusoidea has been proposed recently^2^. More than 1300 extant species of freshwater crabs have been described to date^1^. Freshwater crabs have a pantropical distribution range in the inland water bodies of the continents and adjacent islands, mainly in tropical and subtropical habitats of Neotropical, Palaearctic, Oriental, Australasian, and Afrotropical biogeographic regions^1^. They are among the largest detritivorous macroinvertebrate species^3^ in freshwater ecosystems, where they play vital functional roles in ecological structure^4, 5^.

On the other hand, due to the rapid loss and deterioration of habitats in freshwater ecosystems, especially in the tropics, the survival of many species, including crabs, is endangered. Approximately one-sixth of all freshwater crab species are at high risk of extinction, and approximately one-third are endangered^6^. Most species of endangered crabs are endemic to a restricted-range habitat and survive under the pressure of habitat loss, changing drainage patterns, and water pollution. Therefore, more studies are needed to understand their diversity and to protect endangered populations^6^. This is especially important in Middle Eastern countries, where freshwater ecosystems are under numerous environmental pressures. Iran, in the region, has one of the most diverse varieties of freshwater habitats, but most freshwater studies focus on ichthyofauna conservation^7^. Freshwater studies on freshwater crabs usually focus on taxonomy and species descriptions. In Brandis et al.^8^, seven species of freshwater crabs of the genus *Potamon* have been reported from Iran: *Potamon persicum* Pretzmann, 1962; *P. ruttneri* Pretzmann, 1962; *P. strouhali* Pretzmann, 1962; *P. transcaspicum* Pretzmann, 1962; *P. ibericum* (Beiberstein, 1808); *P. bilobatum* Storch and Turkay, Brandis, 2000 and *P. gedrosianum* Alcock,1909. Later, Keikhosravi and Schubart described a new species, *P. ilam* Keikhosravi and Schubart, 2014^9^, and revalidated *P. elbursi* Pretzmann, 1976^10^. Posteriorly, *P. gedrosianum* was reported in Iran from Zabol (southeastern Iran)^11^.

The identification of the different species in the field, in collections or in labs is relatively challenging due to the similarity and high variation in the morphological characteristics of these species. The lack of taxonomic expertise and field guides makes it difficult to truly evaluate the conservation statuses and diversity of these important members of freshwater ecosystems. In recent years, with the development and proliferation of molecular techniques, one could easily identify different populations, directly or indirectly (i.e., environmental DNA approaches). Molecular approaches present their own set of challenges, one of them being the availability of easily accessible reference databases. In this study, we sampled different populations of freshwater crabs of the *Potamon* genus to (i) identify their taxonomic placement, (ii) evaluate the taxonomic validity of the recognized species using molecular data, and (iii) create a barcode reference for different species inhabiting Iranian freshwater ecosystems.

## Results

**Figure 1:**
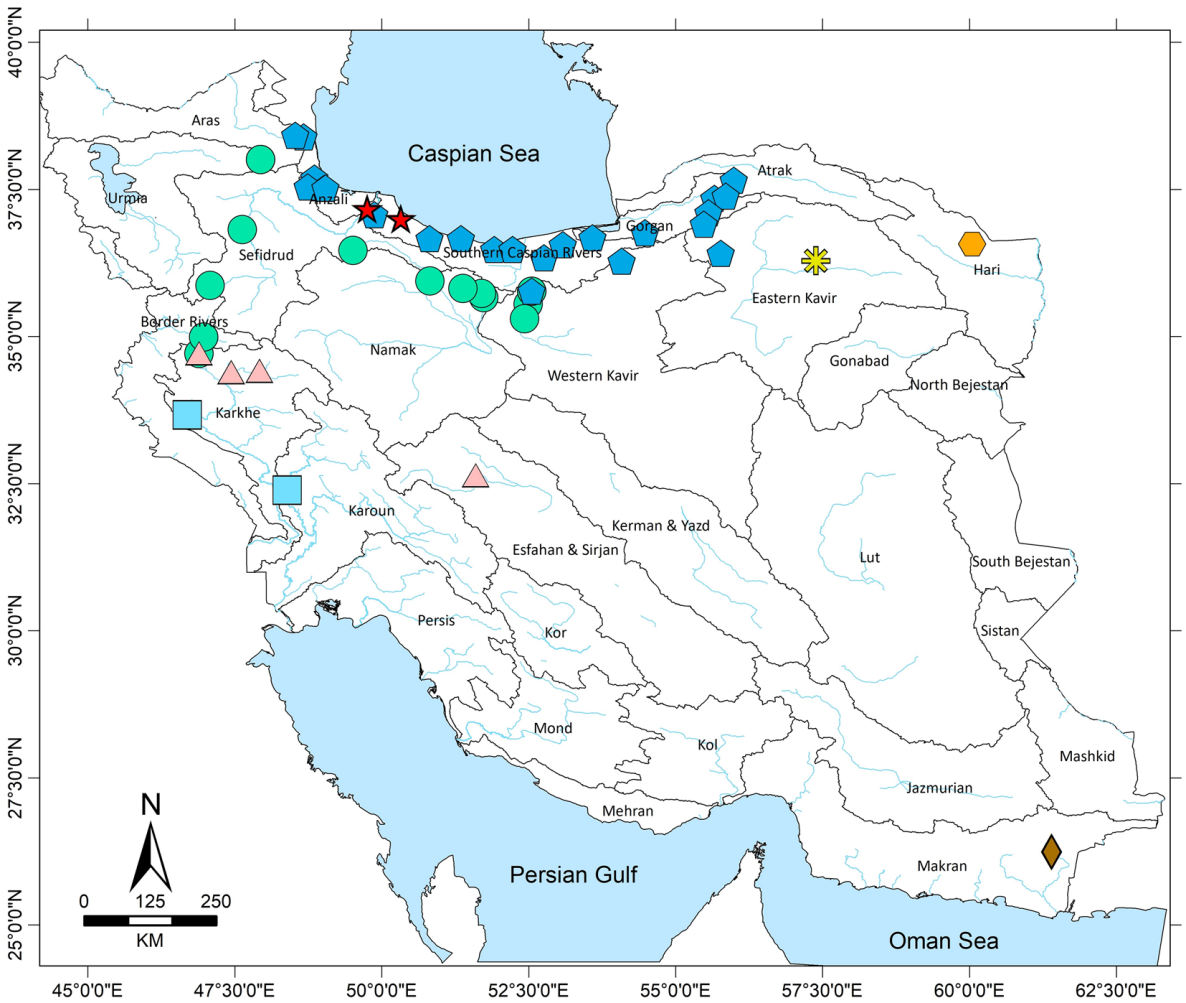
The sampling map of the studied populations. Points marked with an blue pentagon represent *P. ibericum*, red star *P. bilobatum*, green circle *P. elbursi*, pink triangle *P. persicum*, light blue rectangle *P. ilam*, yellow asterisk *P. transcaspicum*, orange hexagon *P. ruttneri* and brown diamond *P. gedrosianum*. The colours used for each species correspond to the same colours used in Fig. 2. The map was created using the software ArcGIS 10.8.1.

**Figure 2:**
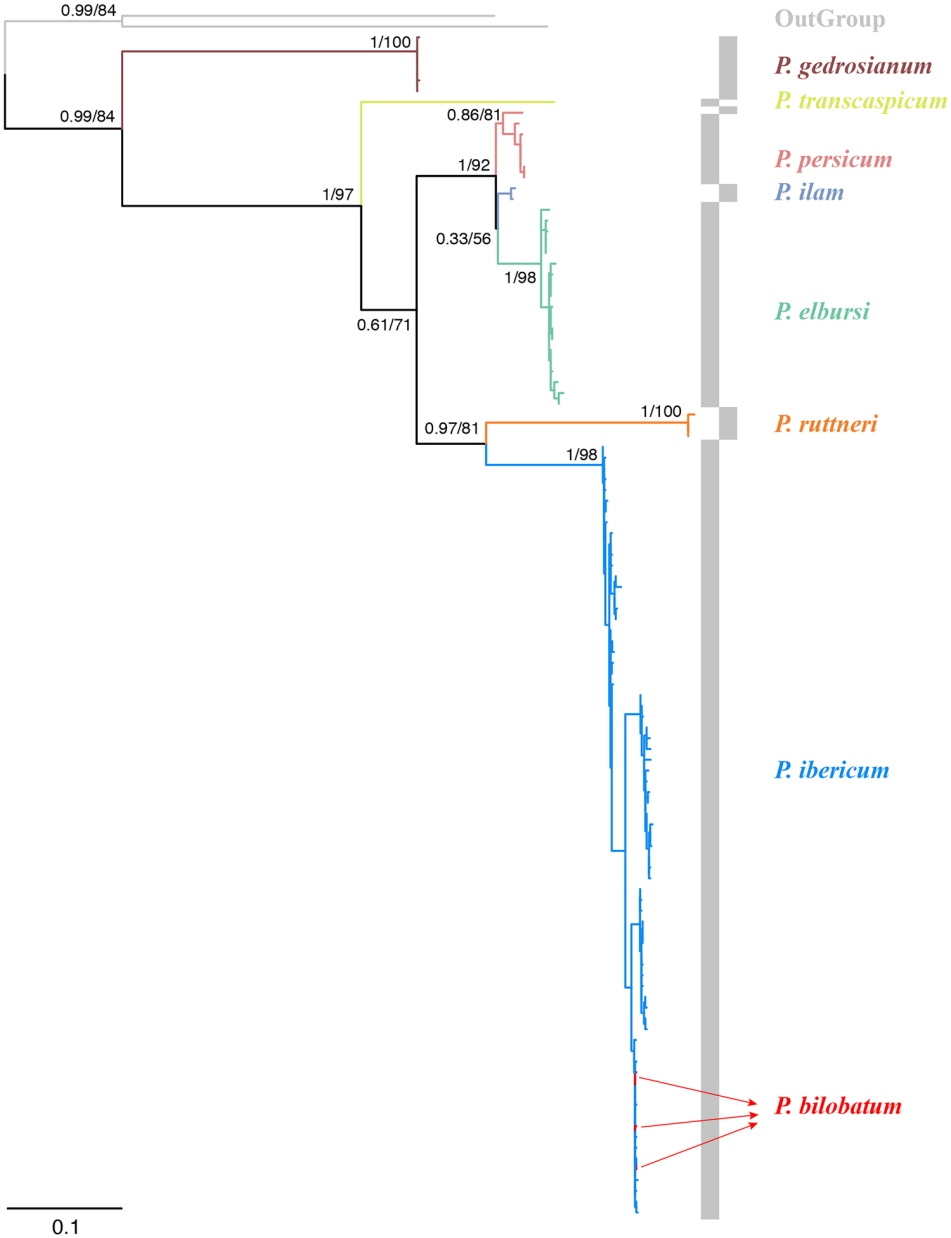
Tree based on the mitochondrial barcode region of Iranian freshwater crabs. Values on nodes represent a-bayes values/bootstrap. The list of taxa is available in Supplementary Table 1. The colours used for each species correspond to the same colours used in Fig. 1.

The final sampling resulted in 110 individuals from six species (Table 1), which covers all the major freshwater bodies of Iran inhibited by this genus (Fig. 1). The final dataset consisted of 923 positions (mean sequence length of 798 bp), from which 205 were parsimony informative, 36 singletons and 682 invariable sites. ModelFinder analysis did not merge any partitions; therefore, each codon position formed an independent partition. The resulting phylogenetic tree (Fig. 2) does not have enough resolution to recover the phylogenetic relationships of the genus. However, each species forms a relatively clear cluster, which helps identify the species boundaries. All species recovered highly supported monophyletics, with the exception of *Potamon persicum*. The only two sequences representing *P. bilobatum* in our study were clustered inside the *P. ibericum* clade, making them unidentifiable from the latter mentioned species. Sequences identified as *P. gedrosianum* were placed with high support as the sister group to all other species of *Potamon* from Iran. *P. transcaspicum* was only represented by a single sequence. Despite having a wide distribution and being overrepresented in this study, *P. ibericum* does not show a clear population structure. The genetic distances observed within each species were highest in *P. ibericum,* with a 2% genetic distance (Table 2). The shortest genetic distance between sister species is 3% between *P. persicum* and *P. ilam*.

**Table 1:** The list of the samples used in this study.

#	Taxa	Locality	GenBank	Reference
1	*P. bilobatum*	Southern Caspian Sea region	MG729765	Parvizi et al.^12^
2	*P. bilobatum*	Southern Caspian Sea region	MG729766	Parvizi et al.^12^
3	*P. bilobatum*	Southern Caspian Sea region	MG729767	Parvizi et al.^12^
4	*P. elbursi*	Mahneshan, Ghezelozan River	HG321389	Keikhosravi & Schubart^10^
5	*P. elbursi*	Tehran, Darakeh, Darakeh R	KF227379	Keikhosravi & Schubart^10^
6	*P. elbursi*	55 km Nw Qazvin, Molali River, Trib To Shahrood	KF227385	Keikhosravi & Schubart^10^
7	*P. elbursi*	Hableroud, Simindasht	LC114291	Unpublished
8	*P. elbursi*	Jajroud, Saiedabad	LC114292	Unpublished
9	*P. elbursi*	Hableroud, Zarindasht	LC114293	Unpublished
10	*P. elbursi*	Jajroud, Kkhajir	LC114294	Unpublished
11	*P. elbursi*	Kermanshah Province	MZ506902	This study
12	*P. elbursi*	Kurdistan Province	MZ506903	This study
13	*P. elbursi*	Kurdistan Province	MZ506904	This study
14	*P. elbursi*	Ghezel Ozan River, Kurdistan Province	MZ506905	This study
15	*P. elbursi*	Ilanjuq, Ardabil Province	MZ506908	This study
16	*P. elbursi*	Ilanjuq, Ardabil Province	MZ506909	This study
17	*P. elbursi*	Ilanjuq, Ardabil Province	MZ506910	This study
18	*P. elbursi*	Kordan, Alborz Province	MZ506913	This study
19	*P. elbursi*	Kolucheh, Kermanshah Province	MZ506914	This study
20	*P. elbursi*	Solehbon, Tehran Province	MZ506922	This study
21	*P. elbursi*	Shirin Sou, Qazvin Province	MZ506925	This study
22	*P. elbursi*	Solehbon, Tehran Province	MZ506926	This study
23	*P. gedrosianum*	Rāsk, Sistan and Baluchestan Province	MZ506937	This study
24	*P. gedrosianum*	Rāsk, Sistan and Baluchestan Province	MZ506938	This study
25	*P. gedrosianum*	Rāsk, Sistan and Baluchestan Province	MZ506939	This study
26	*P. gedrosianum*	Rāsk, Sistan and Baluchestan Province	MZ506940	This study
27	*P. gedrosianum*	Rāsk, Sistan and Baluchestan Province	MZ506941	This study
28	*P. gedrosianum*	Rāsk, Sistan and Baluchestan Province	MZ506942	This study
29	*P. ibericum*	Gorgan, Naharkhoran	KF227380	Keikhosravi & Schubart^10^
30	*P. ibericum*	Southern Caspian Sea region	MG729705	Parvizi et al.^12^
To	–	–	To	–
89	*P. ibericum*	Southern Caspian Sea region	MG729764	Parvizi et al.^12^
90	*P. ibericum*	Solehbon, Tehran Province	MZ506907	This study
91	*P. ibericum*	Sangetab, Mazandaran Province	MZ506911	This study
92	*P. ibericum*	Sangetab, Mazandaran Province	MZ506912	This study
93	*P. ibericum*	Āstāne, Semnan Province	MZ506915	This study
94	*P. ibericum*	Tangrah, Golestan Province	MZ506916	This study
95	*P. ibericum*	Tarzuchu, Gilan Province	MZ506919	This study
96	*P. ibericum*	Vazesht, Gilan Province	MZ506920	This study
97	*P. ibericum*	Tarseh, Golestan Province	MZ506921	This study
**98**	*P. ilam*	Ilam Prv., Shirvavn And Chardavol, Chardavol R	KF227381	Keikhosravi & Schubart^10^
**99**	*P. ilam*	Khuzestan Prv., Dezfoul, Dez River	KF227382	Keikhosravi & Schubart^10^
100	*P. persicum*	Hamadan Province	MZ506896	This study
101	*P. persicum*	Hamadan Province	MZ506897	This study
102	*P. persicum*	Bisotun, Kermanshah Province	MZ506898	This study
103	*P. persicum*	Bisotun, Kermanshah Province	MZ506899	This study
104	*P. persicum*	Bisotun, Kermanshah Province	MZ506900	This study
105	*P. persicum*	Kermanshah Province	MZ506901	This study
106	*P. persicum*	Isfahan, Zayandehrood R	KF227383	Keikhosravi & Schubart^10^
**107**	*P. ruttneri*	Razavi Khorasan Province	MZ506906	This study
**108**	*P. ruttneri*	Abgarm, Razavi Khorasan Province	MZ506917	This study
**109**	*P. ruttneri*	Abgarm, Razavi Khorasan Province	MZ506918	This study
110	*P. transcaspicum*	Khorasan Razavi Prv., Sabzevar, Zardkoohi	KF227384	Keikhosravi & Schubart^10^

GenBank stands for NCBI’s GenBank accession numbers. For the exact distribution of the samples and their GPS coordinates, see the supplementary materials.

**Table 2:** Estimates of average evolutionary divergence over sequence pairs between and within groups.

#	Species	1	2	3	4	5	6	7
1	*P. persicum*	**0.011**						
2	*P. elbursi*	0.051	**0.010**					
3	*P. ruttnerii*	0.128	0.146	**0.004**				
4	*P. ibericum*	0.127	0.138	0.124	**0.020**			
5	*P. gedrosianum*	0.152	0.153	0.168	0.156	**0.001**		
6	*P. ilam*	0.029	0.046	0.128	0.124	0.155	**0.005**	
7	*P. transcaspicum*	0.128	0.143	0.155	0.141	0.159	0.124	**n/c**

The number of base differences per site from averaging over all sequence pairs within each group is shown in the diagonal and is marked in bold. “n/c” is shown in one case because only one sequence was available for it.

## Discussion

At present, the Iranian members of the genus *Potamon* are represented by 9 species in the literature: *P. bilobatum*; *P. elbursi*; *P. gedrosianum*; *P. ibericum*; *P. ilam*; *P. persicum*; *P. ruttneri*; *P. strouhali* and *P. transcaspicum*. Based on our results, we suggest that the taxonomic status of *P. bilobatum* should be studied in more detail, and our study supports the synonymy of *P. bilobatum* with *P. ibericum*. As seen in Fig. 2, both nominal species are indifferent from each other in the tree. This result relies on the sequences of samples identified in other studies^12^, where the paratypes of the *P. bilobatum* have been sequenced. Even if the COI barcode region did separate perfectly the other species studied here, a single marker might not be sufficient to confirm the taxonomy of the genus. Therefore, we believe more specific studies on the subject are needed to resolve *P. bilobatum*’s taxonomic status.

The result of our analyses divides the samples identified as *P. persicum* into two independent lineages, which could be caused by the lack of resolution and support in that part of the tree. This could be improved with a higher sampling size for the populations of this species. On the other hand, the average genetic distance within all samples identified as *P. persicum* was comparable to the average genetic distances within *P. ibericum* samples. This supports the idea that the structure observed in the tree for *P. persicum* corresponds to population structures observable in widespread species and is probably not due to a speciation event. The interspecific and intraspecific genetic distance gap in Iranian members of the *Potamon* genus seems to be a value between 2 and 3% genetic distance.

## Conclusions

In this study, we present the first barcode reference for different populations of potamid crabs inhabiting Iranian freshwater bodies. We evaluated the taxonomic statuses of different described species using molecular data that showed rather high genetic diversity within species. This is a first step to improve the identification of the different species for future studies using molecular techniques. Our results offer an important molecular resource for environmental and conservation studies. We believe these results are especially important these days, as eDNA approaches are becoming an important part of all conservation and biodiversity studies, and these approaches rely strongly on molecular references. Proper species identification is the basis for future studies on the ecology and conservation of these highly susceptible species to climate change.

## Methods

### Taxon sampling

A total of 35 specimens from 19 localities were sampled in this project, covering the main distribution range of the genus in Iran. In addition, all available barcode sequences from Iran in GenBank, a total of 75, were downloaded and included in the study (Table 1 and Supplementary Material). Other available COI sequences (eleven in total) from Iran (accession numbers LN833869-LN833879) were omitted from the study, as they corresponded mainly to the second half of the COI gene, which overlapped very shortly with the barcode region, and the rest of our dataset. These sequences were identified as *P. elbursi,* which is represented in our study by other better suited sequences. To root the phylogenetic tree, the barcode sequences for two other potamid species were downloaded from GenBank, *Socotra pseudocardisoma* (AY803585) and *Johara tiomanensis* (AB290644).

We fixed the specimens sampled directly in absolute or 95% ethanol by injecting them into their body and covering them in jars. The diluted ethanol in the jars was changed multiple times in the first days as it absorbs the water of the samples while dehydrating and, therefore, preserving them. We observed that ethanol injection and multiple changes are crucial to obtain well-preserved DNA quality samples, as other specimens sampled not following this procedure did not amplify successfully in the majority of cases. The samples were deposited in the collections of the National Museum of Natural Sciences of Madrid (MNCN-CSIC).

### DNA extraction and sequencing

Genomic DNA was extracted from a small sample (less than 2 mm in size) of muscle tissue of an ambulatory leg using the DNeasy^®^ Blood & Tissue Kit (QIAGEN, Hilden, Germany). DNA purification was carried out using BioSprint 15 and one 5-tube strip per sample. The DNA was eluted in 200 μl of AE buffer and transferred into a 1.5 ml microtube for long-term storage.

The barcode region of the *cytochrome c oxidase subunit I* (COI) gene was amplified using LCO1-1490/HCO1-2198 forward and reverse primers^13^. Amplification was carried out in a total volume of 12 μl per reaction (1–2 μl template DNA, 1 μl of each primer, 2.75–1.75 μl Milli-Q H_2_O and 6.25 μl of DreamTaq Green PCR Master Mix). After confirmation of successful amplification by electrophoresis, PCR products were purified using Exo-SAP-IT^®^ and sequenced using an external commercial company (Macrogen, Seoul, South Korea) with the same corresponding forward and reverse primers. The obtained sequences were quality checked, trimmed and assembled in Geneious software (Geneious^®^ 10.2.6; Biomatters http://www.geneious.com)^14^. They were aligned with MAFFT^15, 16^ implemented in Geneious using the auto algorithm option. Each alignment was trimmed, manually adjusted, and visually verified to maximize positional homology, taking into account the genetic codes and the translation frames of the protein-coding gene. All the sequences have been deposited in GenBank (Table 1).

### Alignment, phylogenetic inference and species delimitation

The final dataset was aligned using MAFFT implemented in Geneious and screened for sequencing errors. Such poor-quality sequencing errors were found in data downloaded from GenBank and corrected using IUPAC general degenerate nucleotide codes (ex. Gaps resulting in frameshift were replaced with Ns where possible). The maximum likelihood approach was used to construct a phylogenetic tree in IQ-Tree v 2.1.2^17^. The best partitioning scheme and substitution model were found using ModelFinder^18^ as implemented in IQ-Tree (-m MFP + MERGE). For the tree reconstruction, 500 nonparametric bootstraps^19^ were used to evaluate the nodal support (-b 500). To delimit species within our dataset, we used bPTP^20^. For the bPTP approach, the phylogenetic tree was analysed using the online portal (https://species.h-its.org/). The “rooted tree” and “delete outgroup” options were selected, and the number of MCMC iterations was increased to 5 * 10^5^. All other parameters were left in default. Alignment statistics and uncorrected distance matrices were obtained using MEGA11^21^. Species delimitation statistics were obtained using the Species Delimitation plugin^22^ on Geneious.

## Supplementary Information

Supplementary Table 1.

## Data Availability

The genetic sequences produced in this study are deposited in GenBank.
